# Perfusion Pressure Cerebral Infarct (PPCI) trial - the importance of mean arterial pressure during cardiopulmonary bypass to prevent cerebral complications after cardiac surgery: study protocol for a randomised controlled trial

**DOI:** 10.1186/s13063-016-1373-6

**Published:** 2016-05-17

**Authors:** Anne G. Vedel, Frederik Holmgaard, Lars Simon Rasmussen, Olaf B. Paulson, Carsten Thomsen, Else Rubæk Danielsen, Annika Langkilde, Jens P. Goetze, Theis Lange, Hanne Berg Ravn, Jens C. Nilsson

**Affiliations:** Department of Cardiothoracic Anaesthesiology, Heart Centre, Rigshospitalet, University of Copenhagen, Blegdamsvej 9, DK-2100 Copenhagen, Denmark; Department of Anaesthesia, Centre of Head and Orthopaedics, Rigshospitalet, University of Copenhagen, Blegdamsvej 9, DK-2100 Copenhagen, Denmark; Neurobiology Research Unit, Neuroscience Centre, Rigshospitalet, University of Copenhagen, Blegdamsvej 9, DK-2100 Copenhagen, Denmark; Department of Radiology, Diagnostic Centre, Rigshospitalet, University of Copenhagen, Blegdamsvej 9, DK-2100 Copenhagen, Denmark; Department of Clinical Biochemistry, Diagnostic Centre, Rigshospitalet, University of Copenhagen, Blegdamsvej 9, DK-2100 Copenhagen, Denmark; Department of Biostatistics, University of Copenhagen, Øster Farimagsgade 5, DK-2100 Copenhagen, Denmark

**Keywords:** Cardiopulmonary bypass surgery, Cardiac anaesthesia, Ischaemic stroke, Embolic stroke, Postoperative cognitive dysfunction

## Abstract

**Background:**

Debilitating brain injury occurs in 1.6–5 % of patients undergoing cardiac surgery with cardiopulmonary bypass. Diffusion-weighted magnetic resonance imaging studies have reported stroke-like lesions in up to 51 % of patients after cardiac surgery. The majority of the lesions seem to be caused by emboli, but inadequate blood flow caused by other mechanisms may increase ischaemia in the penumbra or cause watershed infarcts. During cardiopulmonary bypass, blood pressure can be below the lower limit of cerebral autoregulation. Although much debated, the constant blood flow provided by the cardiopulmonary bypass system is still considered by many as appropriate to avoid cerebral ischaemia despite the low blood pressure.

**Methods/design:**

The Perfusion Pressure Cerebral Infarct trial is a single-centre superiority trial with a blinded outcome assessment. The trial is randomising 210 patients with coronary vessel and/or valve disease and who are undergoing cardiac surgery with the use of cardiopulmonary bypass. Patients are stratified by age and surgical procedure and are randomised 1:1 to either an increased mean arterial pressure (70–80 mmHg) or ‘usual practice’ (40–50 mmHg) during cardiopulmonary bypass.

The cardiopulmonary bypass pump flow is fixed and set at 2.4 L/minute/m^2^ body surface area plus 10–20 % in both groups.

The primary outcome measure is the volume of the new ischaemic cerebral lesions (in mL), expressed as the difference between a baseline, diffusion-weighted, magnetic resonance imaging scan and an equal scan conducted 3–6 days postoperatively. Secondary endpoints are the total number of new ischaemic cerebral lesions, postoperative cognitive dysfunction at discharge and 3 months postoperatively, diffuse cerebral injury evaluated by magnetic resonance spectroscopy and selected biochemical markers of cerebral injury.

The sample size will enable us to detect a 50 % reduction in the primary outcome measure in the intervention compared to the control group at a significance level of 0.05 and with a power of 0.80.

**Discussion:**

This is the first clinical randomised study to evaluate whether the mean arterial pressure level during cardiopulmonary bypass influences the development of brain injuries that are detected by diffusion-weighted magnetic resonance imaging.

**Trial registration:**

ClinicalTrials.gov, NCT02185885. Registered on 7 July 2014.

**Electronic supplementary material:**

The online version of this article (doi:10.1186/s13063-016-1373-6) contains supplementary material, which is available to authorized users.

## Background

Cerebral injury is a serious complication after cardiac surgery with the use of cardiopulmonary bypass (CPB) [1, 2]. Several preoperative risk factors have been identified, most importantly age, proximal aortic atherosclerosis and a history of neurologic disease [1, 3]. The pathophysiology behind cerebral dysfunction after CPB ranges from reversible cell dysfunction to cell death, causing infarcts.

**Fig. 1:**
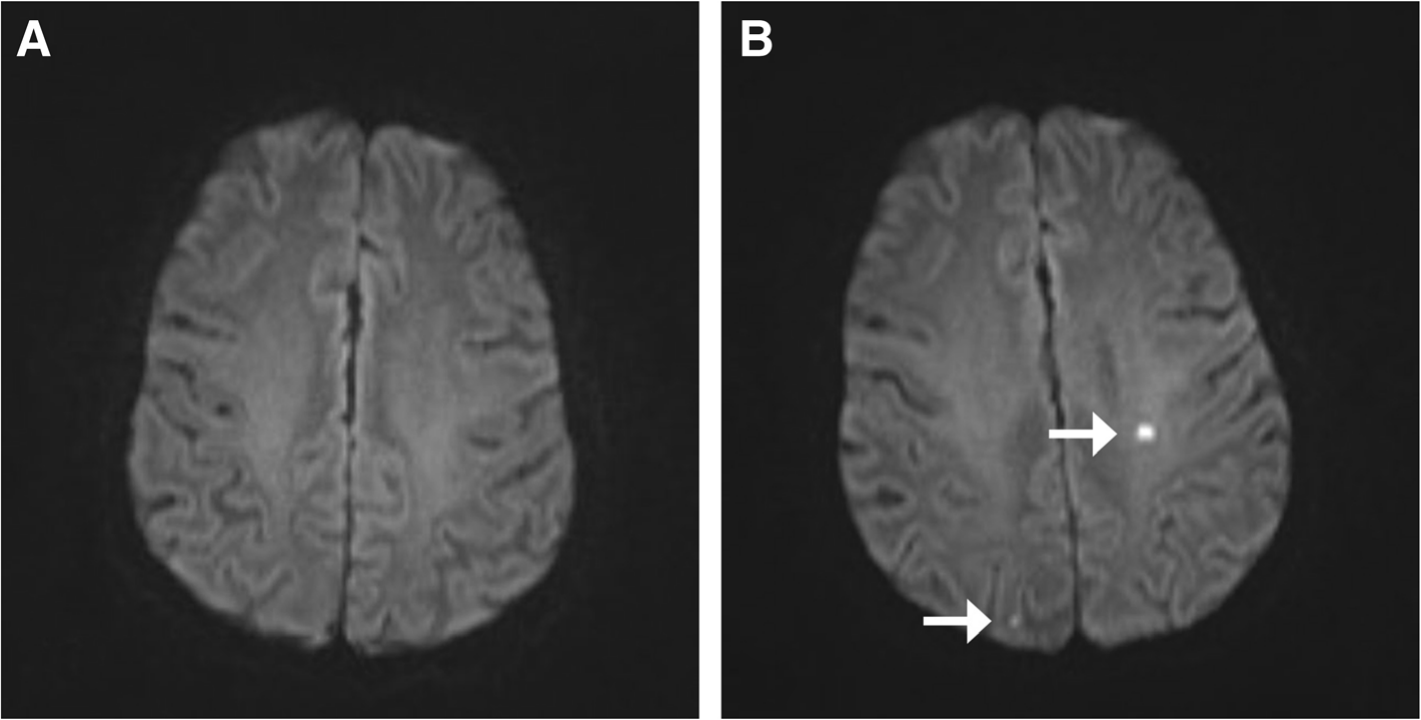
Diffusion-weighted magnetic resonance imaging (DWI) scans of a Perfusion Pressure Cerebral Infarct (PPCI) pilot study participant before (**a**) and after (**b**) heart surgery with the use of cardiopulmonary bypass (CPB). The images reveal two silent cerebral infarcts marked by white arrows

Cerebral infarcts may be caused by emboli, thrombi and/or regional hypoperfusion, with emboli being considered the most common cause. Embolic material can arise from fragments of atherosclerotic plaques, fat, air bubbles and particles from technical equipment [4]. After on-pump coronary artery bypass grafting (CABG) 1.6–5 % of patients experience a stroke, and this risk increases with age [1–3]. When using diffusion-weighted magnetic resonance imaging (DWI), the reported incidence of new ischaemic infarcts in relation to CABG is from 26 to 51 % [5, 6]. Corresponding incidences also have been reported for heart valve surgery [7, 8] (Fig. 1).

Apart from the aetiologies mentioned above, the perfusion pressure during CPB, i.e. the mean arterial pressure (MAP), may contribute to the occurrence of PPCI. The concept of cerebral autoregulation, has been shown to apply not only under regular physiologic conditions between MAP levels of 50–150 mmHg [9, 10] but also during heart surgery with non-pulsatile CPB and moderate haemodilution, where the autoregulation of the cerebral blood flow (CBF) in securing a flow-metabolism coupling [11–14] is limited to a functional range of cerebral perfusion pressure (CPP) between 40 and 120 mmHg [15]. Based on the results from humane clinical studies, Govier et al. [16] and Murkin et al. [14, 17] reported that CBF was independent of MAP down to 30 mmHg or CPP (approximately the MAP minus the jugular venous pressure) of 20 mmHg during hypothermic CPB.

Though debated for many years, the topic of perfusion pressure strategy is still controversial, as many questions remain unanswered. Below the lower limit of autoregulation (LLA), CBF varies directly with CPP, and consequently, the CBF may be pressure-limited, potentially resulting in ischaemic injury. Thus, some have argued a MAP of approximately 50 mmHg during CPB suffices to secure adequate tissue perfusion [18, 19], whereas others have criticized this simplistic application of a classic cerebral autoregulation theory [20, 21] and advocated higher mean pressures (70–80 mmHg) [22] because a right shift of the LLA is expected in atherosclerotic and chronically hypertensive patients [23].

To increase the MAP, a supply of vasoconstrictors is often needed. In a study of 12 anaesthetised swine, O’Dwyer et al. [13] found that an increase of MAP with phenylephrine caused significantly lower values of splanchnic blood flow compared to an increase of MAP achieved by an elevation of pump flow. In general, norepinephrine (α (β)-receptor agonist) and phenylephrine (α_1_-receptor agonist) are considered to have minimal effects on the cerebral circulation and metabolism in healthy subjects, but several studies indicate a change of conditions when the integrity of the blood-brain barrier is compromised [24–26]. In that situation and in the clinical event of an intraoperative stroke or a pronounced inflammatory reaction, norepinephrine is speculated to interact with the α-adrenergic receptors of cerebral vessels, thereby causing vasoconstriction and increased oxygen consumption, which may enhance ischaemia in the penumbra of an embolic stroke or an area of regional hypoperfusion. Physiologically, surgical stress and critical illness are potent endogenous stimuli of the sympathetic nervous system, and when exogenous adrenergic drugs are added, the risk of adverse effects, such as hypercoagulability, tachyarrhythmias, diastolic dysfunction etc., increase as a consequence of sympathetic overstimulation [27].

Until now, only three randomized clinical trials have investigated the importance of MAP during CPB on neurological and cognitive outcomes [22, 28, 29]. In all three trials, a target MAP of 80 mmHg or above in the high pressure group was compared to various levels of control group MAPs. Gold et al. found a significant difference in favour of high pressure with the primary endpoint of major cardiac and neurological morbidity but found no significant difference in the isolated neurological complications. A decade later, Charlson et al. found no difference when comparing a target MAP of 80 mmHg to a custom pre-bypass pressure MAP. In a more recent study, the incidence of neurocognitive dysfunction and delirium was lower in patients with high MAP. These somewhat conflicting observations could be related to the patient selection, methodology or choice of endpoint. For this reason, neuroimaging with identification of newly developed injuries may provide a more reliable estimate of neurological injuries at different MAP management levels, and the present study will be the first to do so in a clinically randomised fashion.

In conclusion, the importance of MAP during CPB on cerebral complications after cardiac surgery is unknown [30]. The task of avoiding a hypotension-induced limitation of flow may come at the price of increasing the risk of cerebral vasoconstriction and hypercoagulability due to the necessary application of vasopressor therapy. Thus, this state of equipoise inarguably justifies the scientific efforts made towards a clarification of benefit versus risk in the application of the different MAP strategies during CPB.

### Aim

The aim of the PPCI trial is to assess the effects of two distinctive levels of MAP during CPB on the development of perioperative cerebral injury in cardiac surgery patients.

## Methods/design

This trial is a single-centre superiority trial with a computer-generated allocation sequence, centralised web-based randomisation and blinded outcome assessment of patients with coronary vessel and/or valve disease undergoing cardiac surgery with the use of CPB in a university hospital in the capital region of Denmark. Patients will be stratified by age (stratum 1: 18–70 years; stratum 2 ≥ 70 years) and by the type of cardiac surgery (stratum 1 - surgery involving the aortic and/or mitral valve; stratum 2 - surgery not involving these valves), and randomised 1:1 to either an increased MAP (70–80 mmHg) or ‘usual practice’ in our institution (typically 40–50 mmHg) during CPB. In both groups, the CPB blood flow is maintained while fixed at 2.4 L/minute/m^2^ body surface area plus 10–20 %, unless the surgical procedure conditions require a transient reduction in flow.

**Fig. 2:**
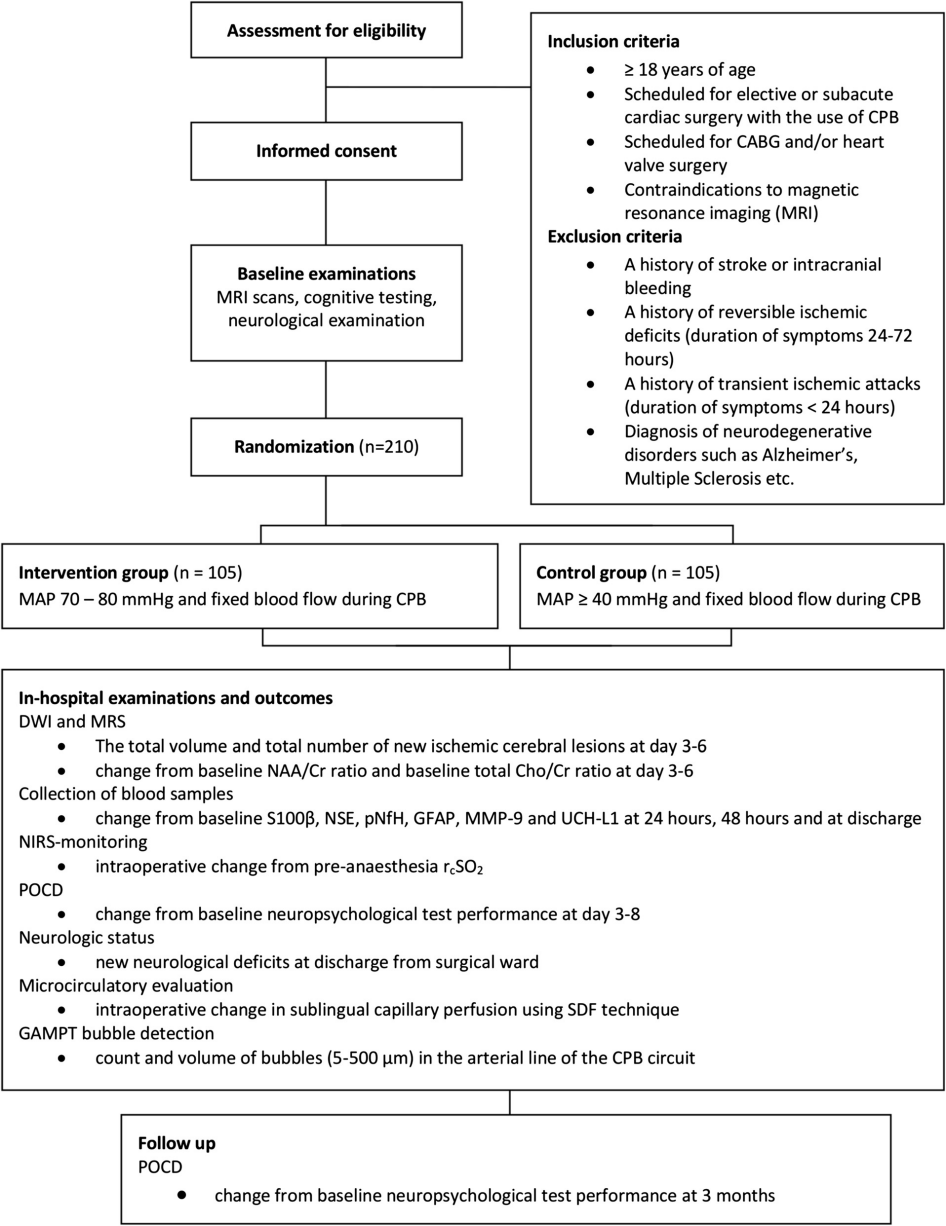
The Perfusion Pressure Cerebral Infarct (PPCI) trial flow chart

Based on the results of a pilot study including 12 patients, a sample size calculation for the main trial was performed. The trial flow chart is shown in Fig. 2. For a populated SPIRIT checklist, please see Additional file 1.

### Hypothesis

If, and to what extent, MAP should be increased during CPB to reduce cerebral complications after cardiac surgery remains controversial because the present evidence is not sufficient to support one perfusion pressure strategy over another, which underscores the need for this trial.

Our hypothesis is that a MAP of 70–80 mmHg with a fixed blood flow during CPB reduces the extent of DWI-evaluated brain injury after cardiac surgery compared to a MAP of 40–50 mmHg during CPB.

### Trial intervention

The trial includes two different groups.

- *Intervention group:* The CPB procedure is conducted according to department guidelines with the modification that MAP is maintained between 70 and 80 mmHg.
- *Control group:* The CPB procedure is conducted in accordance with department guidelines, where MAP is sought to be ≥ 40 mmHg (typically 40–50 mmHg).

CPB blood flow is intended to be equal and fixed in the two groups. The requested MAP level is achieved by intermittent intravenous doses of phenylephrine to a total maximum of 2.0 mg and takes place after that continuous intravenous infusion of norepinephrine up to 0.4 μg/kg/min.

### Inclusion criteria

Patients included in the trial are as follows:

- ≥18 years of age
- Scheduled for elective or subacute cardiac surgery with the use of CPB
- Scheduled for CABG and/or heart valve surgery
- Contraindications to magnetic resonance imaging (MRI)

### Exclusion criteria

Patients fulfilling one or more of the following criteria will not be included:

- A history of stroke or intracranial bleeding
- A history of reversible ischaemic deficits (duration of symptoms 24–72 hours)
- A history of transient ischaemic attacks (duration of symptoms < 24 hours)
- Diagnosis of neurodegenerative disorders such as Alzheimer’s, multiple sclerosis etc.

### Randomisation

Trial coordinators (Anne G. Vedel and Frederik Holmgaard) have 24-hour access to a web-based screening and randomisation system externally developed and supplied by Zenodotus ApS (directed and managed by Dan Høfsten, MD, PhD - CVR-no.: 33 52 41 02 (Danish Central Company Registration no.), Hundested, Denmark) and will, after the entry of stratification variables (age less than 70 years or more than/equal to 70 years; surgery involving the aortic and/or mitral valve or surgery not involving these valves) enrol participants and assign them to either the control or intervention group treatment (Fig. 2a and b). The allocation list is computer generated by Zenodotus ApS, with varying block sizes of four to eight, and allocation will, at all times during the PPCI data collection, be concealed to both the trial coordinators and other project group members.

All patients scheduled for elective or subacute CABG and/or heart valve surgery in our institution for the duration of the recruitment period will be screened for eligibility.

### Primary outcome measure

- The total volume of new ischaemic cerebral lesions (sum in mL), which is expressed as the difference between a DWI scan conducted preoperatively and one conducted on the third to sixth postoperative day.

### Secondary outcome measures

- The total number of new ischaemic cerebral lesions — expressed as the difference between a DWI scan conducted preoperatively and one conducted on the third to sixth postoperative days
- Magnetic resonance spectroscopy (MRS) [31] *—* change from baseline N-acetylaspartate-creatine (NAA/Cr) ratio at days 3–6
- MRS —change from baseline total choline/creatine (Cho/Cr) ratio at days 3–6
- Biochemical markers of brain injury —change from baseline blood concentration of S100ß, neuron-specific enolase (NSE), phosphorylated neurofilament heavy protein (pNfH), glial fibrillary acidic protein (GFAP), matrix metallopeptidase 9 (MMP-9) and ubiquitin c-terminal hydrolase 1 (UCH-L1) [32, 33]at three time points: 24 and 48 hours after surgery commencement and on discharge from the surgical ward
- Postoperative cognitive dysfunction (POCD*)* [34] *—*change from baseline neuropsychological test performance at days 3–8 (i.e. performance at discharge from surgical ward)
- POCD —change from baseline neuropsychological test performance at 3 months
- Near Infrared Spectroscopy (NIRS)—change from pre-anaesthesia regional cerebral oxygen saturation (r_c_SO_2_), measured bilaterally
- Neurologic status — new neurological deficits at discharge from surgical ward.
- Microcirculatory evaluation — intraoperative change in sublingual capillary perfusion evaluated using Sidestream Dark Field (SDF) imaging technique [35] at three intraoperative time points: (1) after anaesthesia induction, before surgery commencement; (2) during CPB; and (3) after CPB during skin closure
- GAMPT bubble detection — count and volume of bubbles (5–500 μm) in the arterial line of the CPB circuit for the duration of the bypass procedure

### Anaesthesia and CPB procedure

During the CPB procedure, we will use a fixed-flow approach (where the pump flow is set at 2.4 L/minute/m^2^ body surface area plus 10–20 %) and the vasoactive therapy regimen described in the trial intervention section. All concomitant medication or treatment interventions will be at the treating clinicians’ discretion.

Our department guidelines on anaesthetic and CPB procedure approaches are as follows in this paragraph: anaesthesia is induced with fentanyl (10 μg/kg), propofol (1 to 2 mg/kg) and cisatracurium (0.1 mg/kg) and maintained with sevoflurane (0.5 % to 3 %) and a continuous infusion of remifentanil (15 to 30 μg/kg/hour). Two peripheral veins, one radial artery and an internal jugular vein are cannulated. Transoesophageal echocardiography and a pulmonary artery catheter are used when considered necessary by the anaesthetist and/or surgeon. The ventilation mode is volume controlled, without the use of positive end-expiratory pressure (PEEP) intraoperatively, and lung recruitment is performed after CPB. After heparinization (350 IU/kg, ACT > 480), an angled arterial cannula (DLP 24 FR, Medtronic Inc., Minneapolis, MN, USA) is surgically placed in the ascending aorta followed by a two-stage venous cannula (36/46 FR, Medtronic) in the right atrial appendage. Normothermic CPB perfusion with non-pulsatile flow is performed using membrane oxygenation with an integrated arterial filter of 25 μm (Affinity Fusion, Medtronic Inc., Minneapolis, USA) and a roller pump (Stockert S5, Sorin Group, Milano, Italy).

For routine procedures, we do not use r_c_SO2 monitoring or arterial line bubble detection [36, 37] in our institution. Accordingly, in this trial, data will be collected only as part of the research protocol. Intraoperatively, we will continuously collect cerebral NIRS data (using Somanetics INVOS 5100C oximeter (Medtronic/Covidien, Denmark)) [38, 39] and save it for later analysis. We will conceal and mute the NIRS monitor during the procedure. Integrating the GAMPT BCC200 (GAMPT GmbH, Merseburg, Germany) into the CPB-machine enables us, through ultrasound Doppler techniques, to detect gaseous microemboli (GME, volume between 5 and 500 μl). In our setup, an ‘arterial’ probe is placed after the oxygenator on a 3/8 inch non-coated PVC tubing line and a ‘venous’ probe is place on a 3/8 inch non-coated PVC tubing line between the roller pump and the oxygenator in the CPB circuit.

### Magnetic resonance imaging protocol

DWI visualises changes in the self-diffusion of water molecules. The technique is highly sensitive for detecting brain ischemia because it enables a distinction between infarction-associated cytotoxic oedema and vasogenic oedema [40, 41].

We will obtain baseline MRI the day before the scheduled surgery and once again between the third and sixth postoperative day using the same 3 Tesla scanner (Magnetom Verio, Siemens, Erlangen, Germany – software NUMARIS/4, version syngo MR B17). Our imaging protocol will include a sagittal T1, an axial T2, a coronal fluid-attenuated inverse recovery (FLAIR) and an axial DWI sequence. The latter sequence will have a slice thickness of 4 mm and a distant factor of 1.2 mm.

When possible (taking factors related to somatic condition, patient compliance and MRI logistics into account), we will expand the imaging protocol and also obtain short-echo-time proton magnetic resonance spectra [31] from two volumes of interest (VOIs) located in parieto-occipital white matter (volume 25 × 25 × 20 mm [3]) and occipital grey matter (volume 21 × 27 × 20 mm [3]), respectively (PRESS, TE 30 ms, TR 3000 ms, 80 acquisitions [42]). This will enable us to evaluate cerebral metabolism, including changes from baseline at the third to sixth postoperative days using the metabolite ratios for N-acetylaspartate/total creatine (NAA/Cr) and total choline/total creatine (Cho/Cr).

### Blood samples

We will collect blood samples at four different time points during the admission: (1) intraoperatively before CPB; (2) 24 hours post-anaesthesia induction; (3) 48 hours post-anaesthesia induction and (4) on discharge from the surgical department. All samples collected will be venous and drawn from either a central venous line or a cubical vein, except for sample number 1, which will be drawn from the catheter in the radial artery. At each time point, a total of 9 mL is drawn and divided between ethylenediaminetetraacetate (EDTA), citrate-coated and heparin-coated tubes. We will centrifuge the samples for 10 min at 3000 rpm while cooling to 4 °C and then extract and store the plasma in polypropylene test tubes at −80 °C until en bloc assaying can be performed.

### Postoperative cognitive dysfunction

The trial coordinators (Anne Grønborg Vedel and Frederik Holmgaard) will evaluate the patients’ cognitive function on three occasions: (1) the day before surgery; (2) the day before discharge from the surgical ward and (3) after 2–4 months. Each time, they will use the ISPOCD test battery [34], which consists of a ‘Visual Verbal Learning test’, a ‘Concept Shifting test’, a ‘Stroop Colour Word Interference test’ and a ‘Letter Digit Coding test’.

As the ISPOCD test battery is developed for patients with an intact cognitive capability, we will conduct a Mini-Mental State Examination (MMSE) to screen for cognitive impairment, i.e. signs of dementia. If a patient has a baseline MMSE score of 24 or less, that patient will not undergo further cognitive testing during the trial.

### Blinding

Patients will be blinded from the group allocation. Additionally, healthcare providers in the intensive care unit and ward will be unaware of the assigned MAP strategy, unless they specifically consult the handwritten anaesthesia report and, based on the continuous registration of MAP data, guess to which group the patient has been allocated. Throughout the trial, we will stress that the staff involved in the experimental setup in the operating room may at no point disclose group allocation.

Since the trial coordinators will conduct screening, patient inclusion/randomization and intervention communication along with MRI scans, POCD tests, blood sample handling and neurological examinations, they cannot be blinded to the assigned MAP strategy. However, assessors of the primary and selected secondary endpoints (DWI scans, MRS and POCD) will be blinded to treatment allocation. Furthermore, blood samples will be labelled with a unique patient number, and personnel carrying out blood sample analysis will not know the group allocation.

The independent trial statistician will also be blinded to the allocation during analysis of both the primary and secondary endpoints.

### Participant withdrawal

Patients may withdraw their informed consent to participate in the trial at any time. If consent is withdrawn on the day of surgery before anaesthesia induction, the trial intervention will be cancelled along with further data registration. Should a patient wish to withdraw their consent to participate in the trial after the surgical procedure has been performed, we will ask the patient to consider participating in some but not all of the trial investigations, in order to collect as much post-intervention data as possible. However, if the patient declines and requests a full withdrawal, no data will be collected. We will report data on all randomized patients and also, to obtain the full sample size, include and randomize additional patients to compensate for missing data regarding the primary outcome measure.

To promote the retention of trial participants, we will – when logistically doable – conduct the 3-month neuropsychological test in the participants’ private homes, should they decline to participate in a follow-up visit at the hospital investigation site due to transportation issues.

### Safety and monitoring

The trial protocol may be suspended for the individual patient at the discretion of the anaesthetist in case of unforeseen haemodynamic challenges that require a different CPB strategy to keep the patient safe.

Missing data on the primary outcome are to be expected, We will obtain an MRI between the third and sixth postoperative day and do expect some missing data on the primary endpoint, since patients must be fully awake and able to complete a 10-min MRI stroke scanning protocol in the supine position with a nasal cannula delivering no more than 3 litres oxygen/min.

There will be no protocolised interim analysis during the trial. We will record severe adverse events (SAE), defined as stroke, acute myocardial infarction, acute kidney injury, gastrointestinal complications (defined as conditions leading to acute explorative laparoscopy/laparotomy) and postoperative bleeding resulting in reoperation, and in addition, mean lactate levels. The trial steering and management committee will monitor and compare the SAE and mean lactate levels between the two groups after inclusion of 50 and 120 patients. Should there be significant differences in frequency of SAE or lactate levels, we will facilitate a critical review of data performed by an external monitoring group, before we continue data collection.

### Statistics

Using the results from the pilot study, the sample size in the present trial has been determined in order to allow for the detection of a 50 % reduction in the primary outcome measure in the intervention as compared to the control group (from 0.18 to 0.09 mL, SD 0.20 mL) at a significance level of 0.05 and with a power of 0.80. The power calculation was based on a t test and showed that 2 × 78 participants would be needed. To compensate for possible non-normal distribution of data, attrition and other causes of missing data, the total number of participants is planned to be 2 × 105.

The primary outcome will be compared between the intervention and the control group and adjusted for stratification [43, 44]. It will be expressed as mean difference with 95 % confidence interval. The analyses will be carried out using multiple linear regression analysis entering randomization and stratification status (age less than 70 years or more than/equal to 70 years; surgery involving the aortic and/or mitral valve; or surgery not involving these valves), as well as preoperative total volume of ischaemic cerebral lesions as independent variables. The primary analysis will be performed according to the intention-to-treat principle [45]. In addition, an unadjusted intention-to-treat analysis and a stratification-adjusted per-protocol analysis excluding patients with major protocol violations will be done.

The neurocognitive tests examine the changes in performance for each of the four test variables used, comparing the baseline/first test session with the second and third sessions. A subtraction of the average learning effect is performed as described by the ISPOCD-group [34]. Patients have cognitive dysfunction when two out of seven Z-scores for individual test variables or the composite Z score is > 1.96.

Intergroup differences in the additional secondary outcome measures will be compared using chi-squared tests with OR and 95 % confidence interval or two-group t tests/Mann Whitney tests, depending on the type of data and their distribution. Intragroup differences (course over time) of blood levels of biochemical markers of brain injury will be examined using repeated measures analysis of variance (ANOVA). Finally, a number of exploratory analyses will be carried out using multiple regression analysis.

If more than 5 % of the cases (10 patients or more) have missing data for any particular analyses and the data can be justified as missing at random, the analyses will be conducted using multiple imputations. The multiple imputation procedure will be based on all available data for that patient and be conducted using the chained equation approach. If data are not missing at random, the analyses will be carried out using available data with appropriate interpretational reservations.

Differences in the primary outcome measure will be considered statistically significant if the *P* value is less than 0.05. Analyses of the secondary outcome measures are considered significant if the *P* values are less than 0.01. Only two-sided tests will be used. Statistical analysis will be performed using R statistical software and the Statistical Analysis System software (SAS Institute Inc., Cary, NC, USA).

### Data registration

Data will be registered in the case report form (CRF) from patient notes (source data) by the trial coordinators. The primary trial coordinator, in cooperation with the primary trial investigator, will establish the trial database by entry of data from the paper CRFs. A data validation will be performed on 10 % of entered data.

The following data will be registered:

Pre-randomization and baseline characteristics:

- National identification number, sex, ethnicity, age at randomization, height in cm, weight in kg, co-morbidities (previous cardiac, lung, neck or brain surgery, previous thoracic irradiation, previous admission for heart failure, stable or unstable angina, myocardial infarction, cardiac arrhythmia, asthma, COPD, chronic treatments for arterial hypertension, hypercholesterolemia, diabetes, cancer, habitual p-creatinine, estimated glomerular filtration rate (eGFR))
- A detailed heart and lung status, including data from coronary angiography, echocardiography and pulmonary function tests
- Results from pre-operative blood samples (standard laboratory values), alcohol, tobacco and medication status

Intra-operative data:

- Cardiac rhythm, heart rate, MAP, central venous pressure, pulmonary arterial pressure, mixed venous oxygen saturation, cardiac output and fraction of inspired oxygen
- Operating, perfusion and aortic cross clamp time, use of aortic side clamp, number of grafts anastomosed to the aorta, plaques in the aorta when described by the surgeon, total volume of phenylephrine and norepinephrine administered, end surgical fluid status, total volume of various inotropic agent administered, use of blow-by CO_2_ in the surgical site, NIRS data and GAMPT BCC200 data

Postoperative data:

- Cardiac status (arrhythmias, pacemaker need > 24 hours after surgery, direct current conversion, postoperative myocardial infarction (according to ESC classification (ref.)), treated pericardial effusion, maximal level of creatine kinase isoenzyme MB (CK-MB) and troponin T (TnT), coronary angiography), neurological status (stroke, symptoms of delirium (that results in acute administration of antipsychotic medication), convulsions), respiratory status (total intubation time, reintubation, pneumonia, treated pleural effusion, need of supplementary oxygen > 2 days), haemorrhage (amount in mL 24 hours post operation, need of transfusions (total volume and type)), gastrointestinal status (ulcer, severe obstipation, ileus, intestinal ischemia), renal status (maximal creatinine during hospital stay, renal replacement therapy)
- Intensive care unit length of stay (ICU-LOS) in hours, readmission to intensive care unit ICU, need and reason for reoperation, total hospitalization time including stay in the department of cardiac surgery, blood samples (standard laboratory values), destination at discharge (home, local hospital or organ specific department)

2–6 months after randomization:

- Cardiac status (New York Heart Association (NYHA) and Canadian Cardiovascular Society (CCS) classification, arrhythmias, persistent pacemaker need, myocardial infarction, heart failure, peripheral ischemia, venous thrombosis), respiratory status (admission to hospital due to pleural effusion, exacerbation and COPD, pneumonia or other reasons for respiratory insufficiency), sternal problems (pain, infection, re-suturing), medication status
- Neurological status (clinical stroke, convulsions, subjective cognitive decline)
- Total days of any form of filtration dependency or haemodialysis dependency
- Survival status obtained from hospital or civil registries
- If the patient is deceased, date of death

One-half and 1 year after randomization:

- Survival status obtained from hospital or civil registries

### Data handling and record keeping

Data will be handled in accordance with Danish Data Protection legislation. All original records (including consent forms, CRFs and relevant correspondences) will be archived at the trial site for 15 years to allow inspection by Copenhagen University or local authorities. The electronic database file will be anonymized and delivered to the Danish Data Archive and maintained for 15 years.

### Ethical considerations and approvals

The trial will be conducted in adherence to the current version of the Helsinki Declaration, the Danish Law on Protection of Personal Information and the National Health Law.

The Regional Ethics Committee of the Danish Capital Region has approved both the pilot study (protocol no. H-2-2012-025) and the main trial (protocol no. H-3-2013-110, July 10 2103), and both pilot study and main trial protocols were also approved by The Danish Data Protection Agency (Data Protection Act, project file no.: 2007-58-0015, local file no.: 30–0805 (pilot study) and local file no.: 30–1434 (main trial).

The trial protocol is registered at ClinicalTrials.gov (NCT02185885).

The Danish Medicines Agency was consulted but found no indication for evaluation and approval of this trial because the trial investigates the importance of MAP during CPB rather than the effect of a medical drug.

Patients will be enrolled only after written informed consent has been obtained. No bio-bank will be formed from the blood samples collected in the trial.

### Publication

Upon trial completion, the main manuscript with trial results, whether positive, negative or neutral, will be submitted to a major clinical journal for peer review and publication.

The trial steering and management committee will grant authorship depending on personal input according to the Vancouver Principles.

### Timeline

**Fig. 3:**
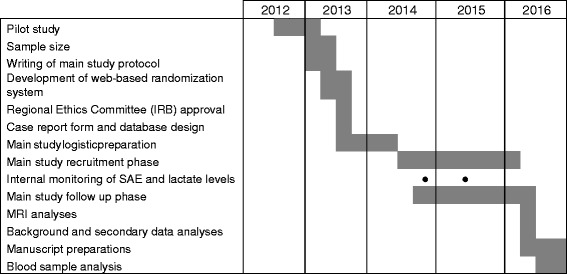
The Perfusion Pressure Cerebral Infarct (PPCI) project timeline chart

A PPCI trial timeline description is presented in Fig. 3.

### Collaborators

This trial is investigator-initiated as an interdisciplinary collaboration between the involved departments at Rigshospitalet, University of Copenhagen, Denmark. Zenodotus ApS (directed and managed by Dan Høfsten, MD, PhD) has developed the web-based randomization system.

### Finances

The PPCI trial is funded by The Danish Heart Foundation and research funds at Rigshospitalet, University of Copenhagen, Denmark. The funding sources have no influence on trial design, trial conduct, data handling and analysis, or publication.

### Perspectives

It is estimated that each year well over 1 million patients worldwide undergo cardiac surgery with the use of CBP [4]. Even though the risk of cerebral injury increases with age, the mean age of our patient population also increases. Thus, it seems more pertinent than ever to uncover the optimal CPB strategy aiming to protect cerebral function in the best possible way. Despite the fact that CPB has been employed and used as standard clinical management during cardiac surgery for more than 50 years, the safety limits regarding cerebral perfusion pressure have never been established. The benefits from a higher MAP versus the potential harmful side-effects from vasopressors should be clearly demonstrated, before a decision on an optimal treatment strategy for MAP during CPB is decided.

## Discussion

The PPCI trial not only investigates DWI-evaluated cerebral lesions, but also aims to correlate these findings to cognitive function, other neurological manifestations, biomarkers of cerebral injury etc., thereby applying a clinical and original perspective on the importance of blood pressure during CPB on cerebral function and monitoring in relation to open heart surgery.

The fact that the primary endpoint is a surrogate outcome is a limitation of the trial. However, conducting stroke and/or mortality studies at a convincingly large scale seems unethical when a more humble, but still relevantly powered, investigation applying neuroimaging can be designed to provide equally important perspectives on the much-debated subject of MAP and the risk of stroke during cardiac surgery with the use of CPB.

## Trial status

The first trial patient was randomised 8 July 2014. We expect to complete patient recruitment in early 2016.

## Additional file

Additional file 1: SPIRIT checklist – recommended items to address in a clinical trial protocol. (DOC 122 kb)

## Abbreviations

ANOVA: analysis of variance
CABG: coronary artery bypass grafting
CBF: cerebral blood flow
CCS: Canadian Cardiovascular Society grading of angina pectoris
Cho/Cr: total choline/total creatine
CK-MB: creatine kinase MB isoenzyme
COPD: chronic obstructive pulmonary disease
CPB: cardiopulmonary bypass
CPP: cerebral perfusion pressure
CRF: case report form
DWI: diffusion-weighted magnetic resonance imaging
EDTA: ethylenediaminetetraacetate
eGFR: estimated glomerular filtration rate
GFAP: glial fibrillary acidic protein
GME: gaseous microemboli
ICU: intensive care unit
ICU-LOS: intensive care unit—length of stay
ISPOCD: International Study of Postoperative Cognitive Dysfunction
LLA: lower limit of (cerebral) autoregulation
MAP: mean arterial pressure
mL: millilitres
MMP-9: matrix metallopeptidase 9
MMSE: mini-mental state examination
MRI: magnetic resonance imaging
MRS: magnetic resonance spectroscopy
NAA/Cr: n-acetylaspartate/total creatine ratio
NIRS: near infrared spectroscopy
NSE: neuron-specific enolase
NYHA: New York Heart Association functional classification of heart failure
PEEP: positive-end expiratory pressure
POCD: postoperative cognitive dysfunction
PPCI: Perfusion Pressure Cerebral Infarcts trial
r_c_SO2: regional (cerebral) oxygen saturation
SAE: severe adverse events
SDF: sidestream dark field imaging
TnT: troponin T
UCH-L1: ubiquitin carboxyl-terminal esterase L1
