# Erratum to: Perfusion Pressure Cerebral Infarct (PPCI) trial - the importance of mean arterial pressure during cardiopulmonary bypass to prevent cerebral complications after cardiac surgery: study protocol for a randomised controlled trial

**DOI:** 10.1186/s13063-016-1753-y

**Published:** 2017-01-12

**Authors:** Anne G. Vedel

**Affiliations:** Department of Cardiothoracic Anaesthesiology, Heart Centre, Rigshospitalet, University of Copenhagen, Copenhagen, Denmark

## Erratum

Unfortunately, the original version of this article [1] contained an error. The listings “Excusion criteria” and “Inclusion criteria” are incorrect. The bullet point “Contractions to magnetic resonance imaging (MRI)” should be listed under "Exclusion criteria" and not under "Inclusion criteria". The correct listing can be found below:

## Inclusion criteria

Patients included in the trial are as follows:

- ≥18 years of age
- Scheduled for elective or subacute cardiac surgery with the use of CPB
- Scheduled for CABG and/or heart valve surgery

## Exclusion criteria

Patients fulfilling one or more of the following criteria will not be included:

- A history of stroke or intracranial bleeding
- A history of reversible ischaemic deficits (duration of symptoms 24–72 hours)
- A history of transient ischaemic attacks (duration of symptoms < 24 hours)
- Diagnosis of neurodegenerative disorders such as Alzheimer’s, multiple sclerosis etc.
- Contraindications to magnetic resonance imaging (MRI)

## References

[1] Vedel A (2016). Perfusion Pressure Cerebral Infarct (PPCI) trial - the importance of mean arterial pressure during cardiopulmonary bypass to prevent cerebral complications after cardiac surgery: study protocol for a randomised controlled trial. Trials.

